# Are we able to have sustainable winter sports events? Main challenges and future directions

**DOI:** 10.3389/fspor.2025.1605544

**Published:** 2025-11-06

**Authors:** Igor Perechuda

**Affiliations:** 1 Department of Management, LUNEX University of Applied Sciences, Differdange, Luxembourg; 2 Luxembourg Health & Sport Sciences Research Institute A.s.b.l., Differdange, Luxembourg

**Keywords:** governance, sustainability, management, event, winter, sports

## Abstract

**Introduction:**

Sustainable practices in winter sports events are increasingly emphasized by policymakers and international associations. However, non-mega event organizers, such as amateur sports clubs and infrastructure owners, often face unique challenges in implementing these practices. This study aims to identify and classify the key challenges and best practices in organizing sustainable winter sports events. Specifically, it investigates: (1) the most significant challenges faced by winter sports organizers (WSOs), (2) whether the perceived importance of these challenges is influenced by WSOs' willingness to adopt sustainability measures, and (3) the most common best practices, their effectiveness in addressing key issues, and remaining gaps.

**Methods:**

A two-step research design was employed. First, a scoping review was conducted to synthesize existing literature on sustainability in winter sports events, focusing on policies, practices, and stakeholder experiences. Second, a questionnaire was distributed using snowball sampling to gather primary data from relevant stakeholders, including sports clubs and federations. The survey explored their experiences with sustainability and event organization. Quantitative data were analyzed using regression analysis and descriptive statistics.

**Results:**

The findings reveal two distinct perspectives within the sector. Policymakers and international associations advocate for standardized sustainability frameworks, while grassroots organizers prioritize operational concerns. Key challenges identified by WSOs include limited financial resources, infrastructure constraints, unpredictable weather conditions, weak public–private partnerships, and natural resource management. The survey also highlighted that WSOs' willingness to implement sustainability practices influences their perception of these challenges. Several best practices were identified, yet some critical issues—particularly financial and infrastructural—remain insufficiently addressed.

**Discussion:**

The study underscores a disconnect between top-down sustainability initiatives and the practical realities faced by grassroots winter sports organizers. While policy frameworks are evolving, their relevance and applicability to smaller organizations are limited. Bridging this gap requires tailored support mechanisms, inclusive policy development, and context-sensitive best practices. Future research should explore mechanisms to enhance collaboration between policymakers and local organizers to foster more effective and inclusive sustainability strategies in winter sports.

## 1 Introduction

While social responsibility and accountability were originally conceived for profit-driven enterprises, these principles have now permeated into nonprofit organizations of all kinds, including those in the sports sector (1, 2). The global trend toward sustainability has infiltrated the sports industry, with growing recognition of the need to mitigate the environmental impact of sporting events (3). This is particularly relevant for winter sports highly dependent on the natural environment and vulnerable to the effects of climate change. Sustainable winter sports events are increasingly feasible through the integration of renewable energy, stakeholder engagement, and innovative management practices. The transition toward sustainability in winter sports is essential, given the environmental impacts and challenges posed by climate change.

In the last 10 years, sustainability has taken on significant importance in the sports industry. Professional sports clubs and national-level sports organizations have increasingly integrated corporate social responsibility (CSR) into their operations, moving beyond traditional philanthropy toward strategic social engagement. CSR in sports is driven both by normative stakeholder expectations and the need for legitimacy in an increasingly scrutinized public environment (4). Especially in European professional football, CSR initiatives are systematically embedded within club operations through foundations, community programs, and environmental policies (5). Many clubs have shifted from *ad hoc* charitable actions to structured CSR programs that align with both business strategies and social objectives, promoting brand value, fan loyalty, and community trust (4, 6). Initiatives often include education, health promotion, social inclusion, and environmental stewardship, illustrating a broader conception of responsibility (7). Environmental sustainability, specifically, has gained prominence as organizations seek to minimize their ecological footprint through green stadium operations, waste management, and renewable energy use (8). Stakeholder perceptions of CSR initiatives greatly influence their success. Authenticity is key: CSR activities perceived as genuine—especially when communicated via credible, familiar sources such as players or local representatives—are more positively received (9, 10). Furthermore, the geographical proximity of CSR efforts matters; local community initiatives generate stronger support compared with distant activities (11). The role of organizational culture and leadership is also critical. Decision-making about CSR is often decentralized, shaped by leaders' personal values and external stakeholder influence rather than purely economic imperatives (4, 12). In many contexts, clubs balance strategic interests with altruistic motives, reinforcing their social role while safeguarding competitive advantage. Overall, CSR has become a fundamental component of strategic management in professional sports organizations, embedding societal engagement within club identity and operations and positioning sports as a platform for broader societal change (5, 8). The same we can observe in the case of the mega sports events’ sustainability approach. There are more and more studies on this topic (1, 13), while non-mega sports and winter sports sustainability challenges remain understudied.

To aid winter sports event managers and organizations in their efforts to enhance the sustainability of their events, published materials, programs, and organizations providing advice in this area have been developed/established. For example, there are several international environmental initiatives by the United Nations Environment Program (14) and an international standard for sustainable event management—ISO 20121 (15). International sports federations also started to be active in this matter. The Fédération Internationale de Ski (FIS) elaborated a strategic plan that incorporated sustainability policy (FIS). The International Olympic Committee (IOC) is recognizing sustainability for winter sports, especially addressing the impacts of climate change on venues and the sport's future. They are working with host cities and regions to establish long-term sustainability policies, encouraging the use of existing venues, and minimizing the Games' carbon footprint (IOC).

The presented study helps fill a gap in the literature by exploring contemporary challenges related to sustainability in winter sports events, specifically in the context of previous research. To this end, we understood how winter sports organizers (WSOs)—defined in this study as institutions involved in organizing non-mega winter sports events, such as national sports federations, winter sports clubs, and sports associations—perceive and address sustainability issues.

While existing studies provide valuable insights, most have concentrated on general public events or large-scale sports and cultural events. There remains a limited focus on non-mega winter sports events, which are crucial for the grassroots development of winter sports. This oversight is particularly significant given that winter sports face unique vulnerabilities to climate change impacts unlike any other sports discipline (16, 17, 115, 116). This article intends to contribute to this emergent area of research and practice by examining the principal challenges faced by winter sports organizers and highlighting underdeveloped areas of inquiry within this context. By adopting the perspective of winter sports organizers, this study identifies and reviews key challenges, aiming to enrich the understanding of sustainability issues in non-mega winter sports events.

In doing so, it contributes to a literature in understanding of current practices in sustainable event management, assisting sports managers with framing “best practice” environmental sustainability strategies for winter sports organizers at the medium and small level. Our study makes three contributions. First, it identifies approaches to sustainability in winter sports events. Second, it throws light on how important sustainability is for WSO as a grassroots organization. Third, by analyzing our dataset through the prism of neo-institutional theory, we provide a better understanding of whether or why WSO have acknowledged their sustainability efforts nowadays.

## 2 Literature background

To identify gaps and narrow aims of this research scoping review of literature was performed. The starting point of this study is the sustainability concept in the sports industry. Following it, we need to disclose the characteristics of winter sports and their contemporary challenges. Finally, a classification of winter sports events research in the context of sustainability was provided to understand why it is still worth researching sustainability in winter sports events management.

### 2.1 Neo-institutional framework for sustainability

Contemporary literature on sustainability in sports and sports events included itself under neo-institutional theory as a theoretical framework (18).

Winter sports organizers are subject to distinct pressures as they attempt to balance environmental sustainability initiatives with their operational and performance objectives. Although some steps have been taken, evidence suggests that sustainability often remains a secondary concern for many organizations (8). Several factors can explain this limited prioritization, including financial limitations, insufficient knowledge or expertise, a lack of commitment to maintaining sustainability efforts, and weak external pressures. McCullough and Cunningham (19) further emphasized that many sports organizations pursue goals that differ significantly from reducing their environmental impact, placing greater emphasis on other operational outcomes (19).

Institutional theory offers a valuable framework for understanding these patterns of environmental behavior (8) and provides insights into why organizations may diverge from sustainable practices despite external expectations (20). Oliver (21) outlined that organizational behavior is shaped by three primary types of pressures: political, social, and functional. Political pressures may emerge from declining performance, a reduction in innovation opportunities, or shifts in stakeholder expectations (21, 22). Social pressures can arise from evolving societal values or internal organizational diversification. Meanwhile, functional pressures are linked to inefficiencies within an organization or intensified competition for resources (21, 22).

In addition to understanding external and internal pressures, it is also useful to consider organizational barriers or evolution to environmental maturity. McCullough et al. (23) proposed the environmental wave typology to describe the progressive stages organizations typically experience as they advance in sustainability practices. Each “wave” captures distinct behaviors and initiatives that signify evolving environmental engagement (23, 24). The first wave is marked by the emergence of sustainability awareness, often prompted by external or internal demands, leading to reactive and generally low-intensity environmental actions rather than integrated strategic initiatives. Examples of early-stage efforts include programs focused on energy savings, waste reduction, and water conservation (23).

Progressing into the second wave, organizations demonstrate increased knowledge of sustainability, formalize their strategic approaches, and often incorporate sustainability principles into their core values. Actions during this stage are informed by positive evaluations of earlier initiatives and represent a more deliberate commitment to environmental practices. The third and most advanced wave occurs when sustainability becomes fully embedded in the organization's strategic planning and daily operations, with sustainable practices becoming normalized and influencing external stakeholders by setting an example (23, 24). This framework highlights how institutional change processes can facilitate the progression toward more environmentally responsible practices in the winter sports sector.

Nuanced application of institutional theory underscores the need for a differentiated analysis of WSOs, whose smaller scale and localized operational contexts distinguish them from their larger counterparts. Following the concept of waves, we could see some other problems in sustainability practice development, such as the gap between sustainability awareness and actual implementation. It may be illuminated through the lens of organizational culture theory (25). Deeply ingrained cultural norms, emphasizing short-term financial imperatives, may inhibit the incorporation of sustainability considerations into organizational practices. That places some attention on the common challenges in medium and small-sized sports organizations, such as financial sustainability.

Neo-institutional theory offers a valuable framework to understand how external drivers such as societal norms, environmental pressures, and industry standards influence organizational change as an adoption of practices across winter sports organizations. Recent studies highlight various approaches to sustainable management in winter sports, showing the effects of regulatory, normative, and mimetic pressures. For instance, Palter and Caraway (26) studied Southern Ontario's private ski clubs and found that climate adaptation investments, such as snowmaking and energy efficiency, align these clubs with industry-wide sustainability trends despite structural differences from public resorts (26).

The implementation of sustainable infrastructure at mega events demonstrates efforts to manage green legacy or green washing, guided by international standards (27, 28). Moreover, the concept of “Twin Transformation,” integrating digitalization and sustainability, has been proposed to enhance organizational performance and promote environmental stewardship within sports management (29). Research on environmental policies and governance identifies both internal and external pressures that drive sustainability efforts, noting that policy entrepreneurs are often critical for championing these initiatives (30). In the study on analyzing challenges and adaptation of sustainable practices in winter sports, we discuss how WSO reflects socio-economic, environmental, and political impact.

### 2.2 Sustainability concept in sports events: key problems in contemporary studies

Sustainability pressure and challenge are visible in wide areas, from various stakeholders and institutions. Nowadays, it is hard to reference just one of these factors. This issue, integral to numerous United Nations Sustainable Development Goals (SDGs), is reflected in challenges such as climate change, carbon emissions, waste management, and the deterioration of natural ecosystems. Furthermore, the depletion of limited resources such as water and energy, coupled with the effects of modern lifestyles and tourism on biodiversity, presents critical concerns requiring urgent and sustained action. The interconnectedness of these factors underscores the pressing need to prioritize environmental sustainability across all aspects of society, including winter sports, physical activity, education, and outdoor recreation.

Academic literature on sustainability in the sports industry has highlighted the multifaceted nature of this issue, with considerations spanning environmental, social, and economic dimensions (31). For instance, recent studies have explored the role of digital technologies in enhancing the sustainability of sports organizations, noting how innovative solutions such as smart energy management systems and digital event platforms can help to reduce the carbon footprint of events (3, 31). Additionally, research has examined how international sports bodies can apply ecological sustainability mechanisms to guide their operations and event management, establishing relevant policies and structures to promote sustainable practices (32).

Moreover, in recent studies, we can find integrating sustainable practices to mitigate the environmental impact of large-scale events, particularly mega events such as the Olympics or World Cups, while fostering community engagement and economic benefits (33, 34).

Environmental sustainability remains a critical issue as sporting events require significant resources, leading to waste generation, energy consumption, and carbon emissions (7). Studies highlight challenges in reducing carbon footprints through energy-efficient facilities, renewable energy usage, and waste reduction programs. However, actual implementation is often inconsistent, with variations across countries and event types (35). Furthermore, greenwashing—the act of misleading consumers about the environmental practices of an organization—continues to undermine true sustainability efforts (36). To overcome these criticisms, we can observe an increasing number of initiatives to secure accountability and transparency [e.g., (37, 38)].

Balancing the economic benefits of hosting sports events with sustainability goals presents a complex issue. Many host cities invest heavily in infrastructure, with uncertain long-term returns, which can lead to debt and underutilized facilities post-event (39). Current studies suggest that while some mega events generate economic boosts, smaller or local events often struggle to achieve sustainability without incurring losses (40). Additionally, the COVID-19 pandemic has intensified financial challenges, compelling event organizers to find cost-effective, sustainable alternatives (41).

Sports events influence social sustainability by impacting local communities. Socially sustainable events prioritize community involvement, equitable access, and cultural preservation (42). However, studies report that local communities frequently experience disruption, displacement, and exclusion, particularly in economically disadvantaged regions (43). Despite efforts to increase inclusivity, the gap between policy and practice remains a barrier to achieving genuine social sustainability in sports events (44).

Implementing sustainable practices at sports events encounters various barriers, including financial limitations, lack of expertise, and insufficient regulatory support (33). Additionally, the inconsistency in sustainability standards and reporting complicates the establishment of universal guidelines, making it challenging to hold organizers accountable (45). Cross-sector collaboration and government policies are increasingly recognized as essential in addressing these obstacles.

### 2.3 Winter sports characteristics

The environmental impact of winter sports and outdoor activities is both significant and multifaceted (46). While much of the existing research centers on alpine skiing and snowboarding, there is limited knowledge about the effects of other winter sports (16, 47). Key contributors to environmental degradation include the construction and maintenance of sports facilities, the hosting of events, and participant-related activities such as transportation. These factors result in energy consumption, resource depletion, waste generation, habitat destruction, pollution, and harm to wildlife (48).

Furthermore, the relationship between sports and the environment is reciprocal—sports activities influence the environment, while environmental changes, particularly climate change, directly affect the feasibility and sustainability of these activities. This dynamic is especially pronounced in winter sports, which depend on snow and cold climates, making them highly susceptible to the effects of global warming (49).

The rising popularity of winter tourism and sports has heightened the strain on delicate ecosystems, particularly in mountainous areas. Despite growing awareness, many winter sports organizations are ill-prepared to address the challenges posed by climate change. The intricate connection between winter sports and environmental sustainability highlights the urgent need for holistic strategies to mitigate environmental, economic, and social impacts.

## 3 Research process

The research process is elaborated into two steps. The first research step is the scoping review, and the second step is based on quantitative data from the survey. The chosen process provided the foundation for the development of an online survey for WSO representatives.

The first step was divided into two content analysis stages. The detailed process of content analysis selection is presented in Table 1. The first content analysis classified groups of topics and problems related to sustainability in winter sports operations. In this step, keywords used in the search were sustainability, winter, and sport. Results not related to any winter sports activity were excluded to focus on problems in winter sports. This first content analysis of the scoping review was performed using open-access academic literature accessible from the Google Scholar database, supported by searching professional reports in Google. The second content analysis in the scoping review was performed to identify the main research aims and key future research directions (mostly repeated among identified sources). Papers were limited by the topic relevance to “sustainability in winter sports events.” In both content analyses, selection was performed till the moment when aims, problems, and limitations started to be repeated. It means that the number of selected papers was saturated (50, 51). The database for that was limited to papers in open access, indexed in Scopus and Google Scholar. Out of 26 papers, 9 of them are referenced in Table 2 [identified aims (problems) and future suggestions were repeated in other collected sources]. Papers chosen were eligible based on the criteria being contemporary, therefore from 2020 to May 2024. Based on the literature review, three research questions were elaborated and presented in the first results section.

**Table 1:** Content analysis process.

Content analysis stage 1: “sustainability in winter sports operations”	Content analysis stage 2: “sustainability in winter sports events”
Records retrieved and screened: 102	Records retrieved and screened: (48 from stage 1+ Scopus results: 19) 67
Records excluded: 56 (not related to winter sports and sustainability)	Records excluded: 41 (not related to events, not academic publications)
Records included: 48 (identified problems started to be repeated, sample saturation was achieved (see Table 2)	Records included: 26
Types of publications included*:* Reports: 8, Academic journals: 40	Types of publications included*:* academic journals (26)
Types of academic journals: nature and science related (14, w/o sports and tourism), sport, sports management, tourism and hospitality related (19), politics (3), management and organization studies (4)	Types of academic journals: nature and science related (7, w/o sports and tourism), sport, sports management, tourism and hospitality related (13), politics (1), management and organization studies (5)
Number of publications in chosen years: 1999 (1), 2002 (1), 2003 (1), 2010 (2), 2011 (1), 2013 (1), 2014 (1), 2016 (2), 2019 (7), 2020 (5), 2021 (6), 2022 (2), 2023 (5), 2024 (8), 2025 (1)	To see the most recent studies based on the number of references from stage 1, we decided to choose publications since 2020, as we observed the topic interest had increased since then.

Source: own elaboration.

**Table 2:** Literature content on sustainability in winter sports events.

Main aims	Future directions
1. Assess ski infrastructure for green energy potential (52). 2. Analyze profitability of renewable energy in ski resorts (53). 3. Explore winter tourism's response to climate change impacts (52, 54). 4. Analyze growth coalition's strategies amidst climate vulnerabilities (54). 5. Achieve carbon neutrality (55) 6. Climate change impacts (53, 54). 7. AI-powered carbon neutrality (56) 8. Interactions between winter sports resorts and the environment (53, 54). 9. Climate change risks for Olympic Winter Games (57) 10. Socially responsible events (55) 11. Promote sustainability and community engagement (58) 12. Examine sustainability awareness (49, 54) 13. Sustainability legacy (55) 14. Sustainability communication (58, 59). 15. Investigate residents’ perceptions (52)	1. Focused on energy policies and regulations (53). 2. Research beyond most common study area: ski resorts and renewable energy analysis (49). 3. Further development of renewable energy in ski resorts (52, 53). 4. Explore barriers to unconventional energy sector growth (53). 5. Identify criteria for sustainability-oriented events (57). 6. Use qualitative methods for a deeper understanding (52). 7. Assess long-term environmental impacts (53, 56). 8. Explore communication strategies in other winter sports events (58). 9. Investigate sustainability practices in different organisational contexts (57). 10. Explore joint collaboration for sustainable participation legacies (54) 11. Investigate long-term planning among stakeholders (54). 12. Explore eco-friendly methods in more sporting events (55). 13. Investigate the shift to an ecocentric paradigm (49). 14. Conduct “in the field” analysis of case studies (49). 15. Investigate long-term impacts of climate change on winter sports (55). 16. Explore practical responses to climate change in sports (49). 17. Investigate athletes’ climate education levels and impacts (55, 59). 18. Address gaps in understanding environmental sustainability in events (49, 55) 19. Explore interdisciplinary approaches to green event management (58).

Source: own elaboration.

In the second step (survey), we used a purposive sampling method in nature (60) and aimed to identify what Patton (61) described as “information-rich” cases relevant to the research topic. This approach is particularly suitable when the goal is to explore specific instances of a phenomenon in-depth, rather than to generalize findings across a broader population (62).

To implement this method, collaboration was sought from winter sports organizations mostly in European countries such as Switzerland, Bulgaria, Croatia, Lithuania, Slovenia, Germany, the Netherlands, Austria, Luxembourg, Spain, Italy, Sweden, Poland, Slovakia, the Czech Republic, Armenia, and France. Selected countries and winter sports organizers from them were based on the assumption that a significant number of these countries have a long tradition of winter sports (63).

A total of 51 winter sports organizers participated in this study, primarily consisting of small to medium-sized organizations that manage non-mega sporting events. Participants were identified using a snowball sampling approach, whereby initial contacts recommended additional relevant organizations. These organizations were subsequently invited to participate via email and phone. Each organization selected a knowledgeable representative—such as a staff member, owner, or member of the organizing committee—who was qualified to respond comprehensively about their respective sustainability initiatives and management practices. Participants were asked to complete a detailed survey comprising 49 items. It should be noted that, although this sample size is suitable for exploratory research purposes, the snowball sampling method used here introduces a potential selection bias, thereby constraining the generalizability of our findings. To enhance external validity and facilitate broader applicability, future research should consider adopting probabilistic sampling techniques or triangulating survey data with additional qualitative or quantitative data sources.

For the purpose of this study, “best practice” was defined as methods or techniques that yield significant positive sustainable outcomes, whether in specific areas or across multiple aspects of an event's operations.

The study's literature review was used to provide the foundation for the development of an online questionnaire for distribution to participants. The questionnaire consisted of three sections: kind of organization, challenges, and best practices. The main part about challenges was elaborated as five-grade scale (Likert) questions to rank them later. In other sections, we elaborated mixed types of questions (closed-ended and open-ended).

The questionnaire was piloted by sending it to a selected group of academics and industry experts from the winter sports Erasmus+ project. These individuals were asked to consider the questionnaire's length, layout, content, ease of completion, and clarity of question wording. Feedback obtained through this means was then used to make amendments to the questionnaire prior to its distribution in June 2024. Data collection took place over a period of 2 months. This process resulted in 51 usable responses (examples of questions in the survey can be found in the Appendix).

Descriptive statistics and regression [Cronbach's alpha, ordinary least squares (OLS)] were employed to analyze and interpret the data using a spreadsheet-based analytical tool. OLS regression served as a supplementary model to evaluate the reliability and consistency of responses. However, with a relatively small sample size (51 observations) and snowball sampling, the application of OLS regression may raise concerns regarding the robustness of the results, particularly due to potential outliers, influential data points, bias, and violations of critical regression assumptions.

To answer the third research question, a content analysis was performed. The researcher analyzed the content of the open-ended question about best practices to become familiar with the data, enabling the identification of emerging themes and concepts. While existing literature offers a framework of potential themes to guide data interpretation, the researchers observe, consistent with Thornberg and Charmaz (64), that themes naturally emerge from the analysis of the text and data itself. Additionally, to support the answer to the third research question, secondary material (content data) about best sustainable practices in winter sports was analyzed (including reports, websites, internal policies, and strategic documents derived from WSOs). So finally, the content analysis was used to classify the most common best practices included two types of materials: open-ended questions in the questionnaire directed to WSOs and secondary material derived from WSOs. The study used a manual attribution analysis. This approach is more sensitive to context than computerized analysis, as both words and their context are identified (65).

It is important to acknowledge that the small sample size in this study would limit its applicability if the goal were to generalize findings to all public events. However, as this inquiry was exploratory in nature, it intended to examine patterns and generate insights rather than establish broad generalizations.

## 4 Results based on scoping review

The scoping review was the first stage of the presented study. It is comprised of two content analyses. Table 3 presents the results of classified groups of topics and problems related to sustainability in winter sports operations. This classification emerged from the search for three key words: sustainability, winter, and sport.

**Table 3:** Main topics and problems studied in the literature.

Topics and problems of sustainability in winter sports events and organizations	Comments and references
1 Impact of winter tourism and sports on climate change 1.1 Traveling and CO2 emissions in winter sports tourism 1.2 Accommodation and ski resort 1.3 Operations and energy consumption in ski resorts	Tourism is often considered as most climate-sensitive sector, with its impacts closely tied to global warming and climate dynamics (46, 48, 66–71).
2 Environmental impact of winter sports 2.1 Natural sites’ protection and soil erosion 2.2 Alteration of biodiversity and decline of wildlife 2.3 Impact on glacier retreat 2.4 Snowmaking impact on water usage and energy consumption 2.5 The impact of light pollution on the environment	Most studies in this field aim to identify how the operation of a winter sports resort affects the environment. The impact is frequently analyzed, with snowmaking and grooming considered the most environmentally harmful practices. Including soils and grasslands, or more broadly, vegetation. Fewer studies have examined the influence of ski activity on wildlife and water quality (47, 49, 72–81).
3 Economic impact of winter sports 3.1 Artificial snow production and facility operations	Cases based mostly on mega events and tourism activity (82–88).
4 Social impact of winter sports 4.1 Implications for long-term winter sports participation and climate awareness 4.2 Collaboration between stakeholders	Mostly analyzed mega events, tourism aspects, and educational needs. In general, it is a quite limited area of study (89–97).

Source: own elaboration based on scoping review.

Based on Table 3 results, we distinguished four groups of sustainability problems in winter sports events and organizations: winter tourism and sports impact on climate change, winter sports environmental impact, winter sports economic impact, and winter sports social impact.

While the pursuit of sustainable winter sports events is promising, challenges remain. The economic reliance on winter tourism in certain regions can hinder the adoption of sustainable practices (54), as growth strategies often prioritize immediate economic benefits over long-term environmental considerations. Additionally, the variability in environmental impacts and management practices across different locations complicates the establishment of universal sustainability standards. Nonetheless, the integration of renewable energy, stakeholder engagement, and innovative technologies presents a viable path toward achieving sustainability in winter sports events.

Key research lines that are related to sustainability in winter sports events are presented in Table 2.

Based on that, we can say there is limited focus on medium and small winter sports organizers. Not enough focus on aspects other than the environmental aspects of sustainability. Authors in analyzed papers identified limitations of the identification of best practices [e.g., (49, 55)]. In response to this literature’s limitations, the study focused more widely on the sustainability idea than just environmental aspects and elaborated three specific research aims constructed as research questions:

What are the most significant challenges faced by WSOs (non-mega event organizers)?
Is the importance of these challenges influenced by WSOs’ willingness to implement sustainability practices?
What are the most common best practices, which of these address the main problems, and which problems still need to be covered?

## 5 Results of questionnaire analysis

The Results section is divided into three parts. The first one related to challenges classification and understanding, and the second one focused on winter sports sustainable practices. The third part is a discussion and comments on the obtained results in reference to the assumed goals.

### 5.1 Winter sports challenges for event organizers

The first stage of results was the identification of key issues from WSO perspectives. To check how important problems related to sustainability are, we elaborated a set of problem items for WSO identified in the literature. Asking them to assess these sets enables us to understand the significance of sustainability from their perspective.

The internal consistency of the questions among all 16 items, measured by Cronbach’s alpha, is approximately 0.88. This value indicates a high level of reliability, suggesting that the questions are consistent in measuring a similar underlying concept. An additional test was performed to check if there is any question affecting the consistency score. Following that, Cronbach's alpha was recalculated for the sample when one of the questions was removed. The value of Cronbach’s alpha varied between 0.8719 and 0.8830.

Skewness reveals that respondents tended to consider infrastructure, human resources, and financial stability as relatively significant challenges (negative skewness). In contrast, environmental issues such as carbon emissions or sustainable supply chains were seen as somewhat less urgent or less critical (positive skewness). Kurtosis shows that respondents generally varied in their opinions, without a strong consensus around any particular rating, as shown by consistently negative kurtosis values.

ANOVA (*F*-statistic: 4.4977) and pairwise *t*-tests confirmed statistically significant differences in the average Likert scale ratings across the 16 challenges (ANOVA *p* < 0.0001). This validates the ranking based on mean scores (Table 4). The top five challenges identified by respondents, based on average ratings, are as follows:

var5: financial resources and stability (mean = 3.63)
var1: infrastructure (outdated, maintenance needed, etc.) (mean = 3.59)
var7: weather conditions (mean = 3.49)
var6: human resources (mean = 3.20)
var12: sustainable snow management (mean = 3.18)

**Figure 1:**
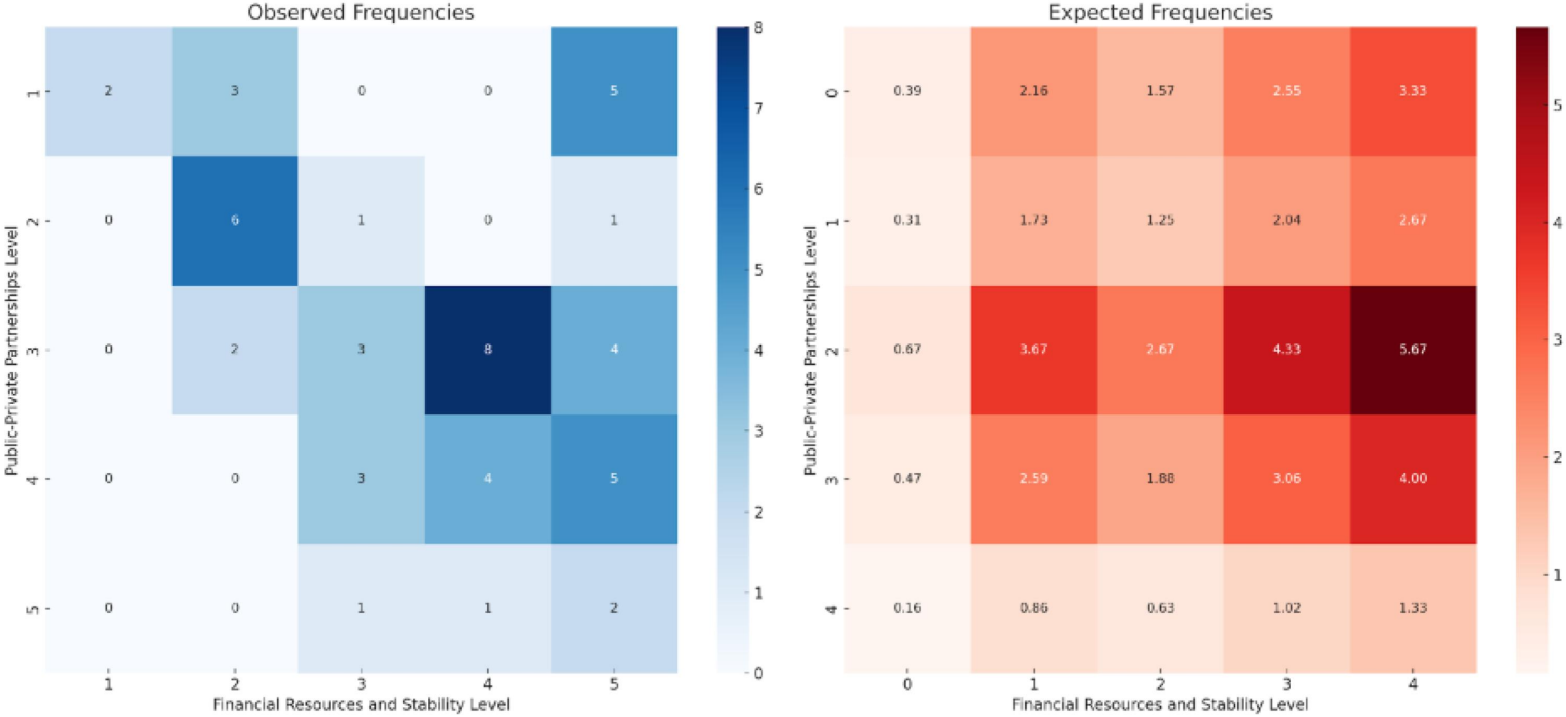
Frequency analysis of finances vs. PPP. Source: own elaboration.

The item analysis of “financial resources and stability” shows that this is the biggest concern among responders. Approximately 60% of them assessed it as important or of high importance. Among the identified top five challenges, we checked if they were somehow dependent or biased on the chosen challenges mentioned as well in the literature. Therefore, financial resources and stability were compared with item 15, public–private partnerships (PPP) (see Figure 1). We see here a statistically significant association between the PPP and financial resources and stability.

**Table 4:** Descriptive statistics of challenge items.

Items (challenge -> question)	*n*	Mean	SD	Min	Max	Median	Skewness	Kurtosis
1. Infrastructure (outdated, maintenance needed, etc.)	51	3.59	1.42	1	5	4	−0.35	−1.46
2. Natural resource usage	51	3.16	1.35	1	5	3	−0.21	−1.11
3. Sustainable transportation	51	3.04	1.39	1	5	3	−0.21	−1.49
4. Regulations	51	2.84	1.29	1	5	3	−0.04	−1.39
5. Financial resources and stability	51	3.63	1.26	1	5	4	−0.28	−1.21
6. Human resources	51	3.20	1.23	1	5	3	−0.38	−1.06
7. Weather conditions	51	3.49	1.57	1	5	4	0.10	−1.52
8. Nature protection	51	2.59	1.30	1	5	3	0.08	−1.29
9. Sustainable supply chain	51	2.29	1.22	1	5	2	0.38	−1.23
10. Waste reduction	51	2.57	1.28	1	5	2	0.22	−1.28
11. Carbon emission of the events	51	2.45	1.30	1	5	2	0.23	−1.53
12. Sustainable snow management	51	3.18	1.60	1	5	3	0.36	−1.26
13. Social issues such as accessibility for people and equality	51	2.71	1.30	1	5	3	0.00	−1.56
14. Decrease in practicing athletes	51	3.12	1.26	1	5	3	−0.16	−1.02
15. Public–private partnerships	51	2.84	1.22	1	5	3	−0.32	−1.16
16. Electricity consumption	51	2.90	1.40	1	5	3	−0.08	−1.45

Source: own elaboration.

Although PPP is considered to have a low importance level based on answers, it is associated with answers given to financial stability. The analysis for “infrastructure (outdated, maintenance needed, etc.)” shows a varied level of concern among respondents. But most of them assessed it as important and very important. At the same time, we can say that infrastructure in organizing winter sports events seems as one of the main concerns. This is in line with our literature review, where identified problems were associated with the ergonomics of infrastructure. In the literature review, it is mentioned that the main researched sustainability problems are associated with energy management, accommodation. At the same time, activities within PPP are one of the development factors for sports infrastructure (98).

**Figure 2:**
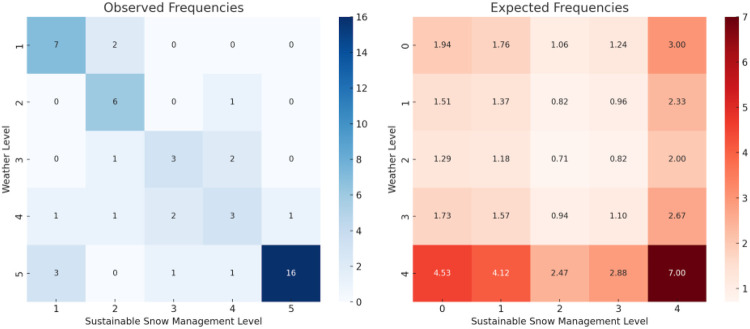
Frequency analysis of weather vs. sustainable snow management. Source: own elaboration.

Another challenge that was mentioned in the literature is the “weather conditions.” In the literature, it is connected to another challenge we have in the survey “sustainable snow management”. Both of them are in our top five challenges. “Weather conditions” presents us a high concern of the problem by more than 40% of responders. Based on additional analysis between weather importance level and sustainable snow management, we see these problems as associated with each other based on the graph (Figure 2).

It can mean that responders may not directly see sustainability as the top challenge, but they identify proxy effects.

The highest ranked challenges overall are related to financial stability, infrastructure, and weather conditions, all of which received the highest average ratings. Environmental issues, such as waste reduction and carbon emissions, were rated lower in comparison with financial and operational challenges.

In the next step, we wanted to additionally check the reliability of the delivered answers. So we verified if given answers about challenges are somehow impacted by declared willingness to implement sustainable practices (variable 1: “var1”) and elaboration of impact report by WSO (variable 2: “var2”). var1 is based on the question with a Likert scale, and var2 is a dummy variable based on the answer given in the questionnaire (yes/no). These two variables were considered as independent variables and compared with the given answers about the main challenges (16 variables, items), which are dependent variables. It delivered us 16 models based on 16 challenges from the survey. OLS regression was performed to provide answers. We see that several models demonstrated statistical significance. The model for sustainable transportation (var3) showed significance with an *R*^2^ of 0.097, and passed both the normality (Shapiro–Wilk *p* = 0.0238) and homoskedasticity (Breusch–Pagan *p* = 0.1811) tests. The model for financial resources and stability (var5) had an *R*^2^ of 0.188 and passed diagnostic checks (Shapiro–Wilk *p* = 0.0205; Breusch–Pagan *p* = 0.8067). Similarly, models for human resources (var6), weather conditions (var7), nature protection (var8), sustainable supply chain (var9), carbon emissions of events (var11), sustainable snow management (var12), and social issues such as accessibility and equality (var13) were statistically significant, with *R*^2^ values ranging from 0.091 to 0.348. All significant models underwent diagnostic testing, confirming the validity of residual assumptions. This regression analysis allows us to address the second research purpose of the study (Table 5).

**Table 5:** Full regression analysis between 16 challenge items and var1 and var2.

Dependent variable	Intercept Coef.	Intercept *P*-value	var1	var1 *P*-value	var2	var2 *P*-value	*R* ^2^	Shapiro–Wilk *P*-value	Breusch–Pagan *P*-value
var_1infrastructure	3.02	0.00	0.20	0.28	−0.77	0.11	0.06	0.00	0.02
var_2naturalresources	1.93	0.01	0.32	0.07	−0.20	0.66	0.07	0.04	0.39
var_3sustainable transportation	1.55	0.03	0.39	0.03	−0.20	0.66	0.10	0.02	0.18
var_4regulations	2.03	0.00	0.24	0.17	−0.41	0.35	0.04	0.05	0.22
var_5financial resources and stability	2.65	0.00	0.34	0.03	−1.26	0.00	0.19	0.02	0.81
var_6human resources	2.63	0.00	0.21	0.19	−0.88	0.03	0.09	0.01	0.71
var_7weather conditions	0.60	0.37	0.84	0.00	−1.42	0.00	0.35	0.22	0.53
var_8nature protection	1.18	0.07	0.36	0.03	−0.10	0.82	0.10	0.19	0.11
var_9sustainable supply chain	1.01	0.10	0.33	0.04	−0.02	0.96	0.10	0.02	0.00
var_10waste reduction	1.90	0.01	0.21	0.22	−0.56	0.20	0.04	0.06	0.96
var_11carbon emission of the events	1.16	0.08	0.38	0.02	−0.78	0.07	0.12	0.01	0.12
var_12sustainable snow management	1.41	0.08	0.49	0.02	−0.60	0.26	0.11	0.01	0.21
var_13social issues	1.36	0.04	0.37	0.03	−0,36	0.40	0.09	0.02	0.05
var_14practicing athletes	3.18	0.00	0.03	0.87	−0.57	0.19	0.04	0.07	0.82
var_15public private partnerships	2.75	0.00	0.00	1.00	0.32	0.46	0.01	0.01	0.85
var_16electricity consumption	1.75	0.02	0.32	0.09	−0.40	0.40	0.06	0.14	0.08

Source: own elaboration.

In Table 6, we can find a list of models that were identified as significant with *p* < 0.05.

**Table 6:** Regression check by willingness to implement and elaboration of the impact report.

Dependent_Variable	Independent_Variable	Coef.	*P* > |*t*|
var_3_transportation	var1_will_sus_practices	0.39	0.03
var_5_financial	var1_will_sus_practices	0.34	0.03
var_5_financial	var2_impact_report	−1.26	0.00
var_6_human_resources	var2_impact_report	−0.88	0.03
var_7_weather	var2_impact_report	−1.42	0.00
var_8_nature_protection	var1_will_sus_practices	0.36	0.03
var_9_supply_chain	var1_will_sus_practices	0.33	0.04
var_11_carbon_emission	var1_will_sus_practices	0.38	0.02
var_12_sustainable_snow_management	var1_will_sus_practices	0.49	0.02
var_13_social_issues	var1_will_sus_practices	0.37	0.03

Source: own elaboration.

We can observe that some of the challenges addressed were significantly supported by the willingness to implement sustainable practices or provide impact reports by WSO.

We identified a positive and significant correlation to such sustainability challenges as follows: *social issues*, *carbon emission*, *nature protection*, *supply chain*, *sustainable transportation*, *snow management*, and *weather conditions* explained by *willingness to implement sustainable practices*. It raises a question whether the given regressions could be explained by knowledge level and awareness about sustainable practices. Seeing as well low average level of importance among these challenges, one of the practical implications could be to raise awareness and knowledge level among WSO. There are some studies in wider sports contexts confirming that building up sustainability awareness results in higher applicability and future prospects of the sports organization (99, 100).

Additionally, all significant models based on the *publishing impact report* are negatively correlated with the importance of challenges such as *financial stability*, *human resources (HR)*, and *weather conditions*. It may be a result of the maturity of chosen WSOs which are developed enough to take care of these challenges previously and now can focus on sustainability actions.

Reminding answer to the first research question, we see that sustainable challenges are not at the top of all challenges that WSO face. Full regression analysis is in the Appendix.

### 5.2 Best practices in sustainable winter sports events

The last part of the study aimed to identify the most common best practices and evaluate their usefulness in relation to previously identified challenges. Based on open-ended questions directed to winter sports organizers (WSO) as well as a comprehensive review of secondary materials provided by these organizations, a classification of sustainable winter sports practices was performed. Eight distinct categories emerged from this analysis. These categories include practices to address air pollution (first), such as measurement and control tools and strategies to reduce transportation carbon footprints through initiatives such as shared transport, price reductions for train travel, collective transportation systems, electric vehicle (EV) charging points during events, event scheduling aimed at reducing travel distances, cooperation with local public transportation, use of drones instead of helicopters for filming, and sourcing local suppliers to minimize transportation impact.

Regarding the second group—social aspects and governance—identified best practices included educational initiatives promoting safe sports practices and responsible mountain behavior through workshops and seminars held both during and between events. Engagement of volunteers, raising sustainability awareness among athletes, National Sport Associations (NSAs), and the broader winter sports community, collaboration with local research centers to identify sustainable solutions, promoting winter sports accessibility within local communities, developing youth programs for skiing and skating, advocating responsible recreation in popular natural sites, enforcing sustainability policies within sports associations and federations, and implementing transparent and reliable sustainability reporting standards were also noted.

Practices for compensation and offsetting (third group) involve reinvesting a percentage of turnover into sustainable initiatives, emphasizing transparency and reliability, and collaborating with trustworthy external partners verified by NSAs.

In the area of energy (fourth group), innovative technologies were highlighted, including wind power generation at high altitudes, hydropower integrated with artificial snow networks, and geothermal energy centers.

Effective snow and ice management (fifth) practices include the production of snow with reduced water use by adding special minerals, creating artificial snow at higher temperatures, implementing snow storage methods, reusing water from ice production for ice rinks, employing heat pumps in snowmaking, and using heat from nearby centers, such as geothermal sources, to produce snow.

Equipment management (sixth) focused on promoting sportswear circulation through rental services instead of purchasing equipment for short seasonal use. Nature and environmental protection (seventh) practices involved adjusting event schedules in coordination with environmental specialists to minimize impacts on local wildlife, aligning winter sports centers' operating times with environmental conditions, and monitoring Alpine lakes.

Finally, waste management strategies (eighth group) highlighted include efficient water management and recycling systems, reduction and recycling of waste and plastics through reusable items, effective food management, sourcing from local food suppliers, and donating used sports equipment and items to local community associations.

Among these groups, we have solution well established with WSOs’ deep experience, and we classified solutions at a very initial stage, not answering the main problems. Table 7 classifies chosen groups of solutions that were well-saturated and developed or underdeveloped. This table was elaborated based on the content analysis of open-ended questions and documents delivered by WSOs.

**Table 7:** Development of classified solutions.

Well-developed solutions (*n*, quantity of practices identified by responders and content analysis)	Underdeveloped (*n*, quantity of practices identified by responders and content analysis)
Waste management (*n* = 4)	Nature and environmental protection (*n* = 2)
Snow and ice management (*n* > 10)	Equipment (*n* = 2)
Energy (*n* = 4)	Compensation and offsetting (*n* = 2)
Social aspects and governance (*n* > 10)	Air pollution (*n* > 10) (transportation problem)

Source: own elaboration.

## 6 Discussion

Regarding the first research question on challenges, the findings were somewhat unexpected. Despite the widespread movement and policy emphasis on sustainability, traditional challenges continue to be at the top of the ranking (Table 4). The top five “big issues” for winter sports organizations (WSOs) include infrastructure, finances, and human resources. Only sustainable snow management and weather conditions, as proxies for environmental challenges, are explicitly related to sustainability.

Previous research in sports management utilizing institutional theory (18) has demonstrated that sports organizations often engage in socially responsible behaviors as a mechanism to maintain legitimacy in response to external institutional pressures. However, extant literature often focuses on professional sports leagues, national associations, regional clubs, national Olympic committees, and mega events, all of which operate under markedly different institutional conditions compared with small and medium-sized winter sports organizations (WSOs).

From an institutional theory perspective, WSOs are embedded in less formalized and less scrutinized environments. In the absence of binding international sustainability regulations and limited visibility among global stakeholders, these organizations are subject to fewer explicit coercive pressures to adopt sustainable practices. Nevertheless, institutional theory posits that WSOs may still encounter normative and mimetic pressures originating from local communities, national federations, athletes, media, and sponsors, which subtly influence their sustainability behaviors.

Furthermore, the study's results align with the tenets of institutional logic. As suggested by Oliver (21), organizational behaviors often reflect institutionalized norms rather than active strategic choices. Thus, it is plausible that the observed gap in WSOs' sustainability engagement has itself become institutionalized within their operational frameworks. This observation invites critical reflection on how WSOs might leverage their latent capacity to advance sustainability initiatives more effectively (101).

It is important to acknowledge that winter sports activities such as skiing are inherently associated with environmental impacts. Practices such as artificial snow production and excessive water consumption are deeply embedded in the daily operations of winter sports events (102, 103). Although sports managers and personnel are well-positioned to contribute to mitigating these impacts, it is unrealistic to expect them to unilaterally resolve complex environmental challenges (104). A comprehensive, strategic approach is recommended—one that accounts for available organizational resources, competencies, and meaningful stakeholder engagement (105–107).

Such an approach should include long-term strategic planning, the cultivation of internal and external stakeholder partnerships, and collaboration with specialized environmental organizations to maximize collective impact. Additionally, the findings of this study align with the conceptual framework of “waves of environmentalism” proposed by McCullough et al. (108), which conceptualizes sustainability engagement as a dynamic, cyclical process characterized by periods of advancement and regression. While the current study offers a cross-sectional view, future longitudinal research would be instrumental in capturing how WSOs evolve in response to sustainability pressures over time.

Regarding the second research question, the study reveals that mature organizations (those that have already implemented impact reporting) show a negative correlation with sustainability challenges. Such organizations have managed traditional problems effectively, enabling them to focus more on sustainability initiatives. It is somehow in line with the literature review. Previous studies on sustainability in winter sports events often focused on mega, giga events (see Table 3). It raises a question for future research: Is there a correlation between organizational maturity and sustainable maturity in WSOs or even sports organizations broadly? In non-sports organizations, this kind of study was already performed deeply (109, 110), but it seems that from the sports perspective, it is a gap to fill.

Referring to findings for the third research purpose (Table 7) in the literature, we observe two notable surprises: one positive and one negative. The content analysis of the literature (Table 4) reveals that studies have already explored social aspects and governance issues in winter sports. In our study, based on the analyzed materials and their content, we found that practices in these areas are developed. This is further supported by survey responses, where social issues are ranked low among the challenges. It may mean the WSOs do not need to pay too much attention to solve this challenge because they are already used to it. Particularly, non-mega event organizers tend to have close relationships with their regional stakeholders.

The negative surprise, however, is the underdevelopment of air pollution practices in the data we analyzed. This is not due to a lack of practices, but rather because the existing practices are insufficient to address the problem effectively. According to studies cited in the literature review (Table 3), transportation is often identified as the most climate-sensitive sector, with its impacts closely tied to global warming and climate dynamics. Thus, the solutions currently provided, along with measures such as progressively limiting access to winter sports regions, are inadequate.

Cross-sector comparisons offer valuable insights for potential adaptation. In European professional football, stadiums have implemented environmental management systems that include low-emission transport options and energy-saving infrastructure to reduce air pollution during matches (111). The World Athletics has also promoted an air quality project for monitoring and traffic reduction strategies during events (112). Furthermore, environmental justice frameworks in sports management emphasize the need for inclusive air quality strategies that consider the unequal exposure to pollution among different communities (113). Finally, research on tourism and air quality highlights how poor air conditions can deter attendance and reduce destination competitiveness, reinforcing the importance of proactive air pollution mitigation during large-scale events (114).

Finally, returning to the literature review on key issues in sustainable winter sports, one area highlighted in this study is snow and ice management. This topic is extensively covered in the literature (see Table 3, points 1.3, 2.4, and 3.1) and is also among the top five issues identified in the study (Table 4). Other sustainability-related challenges ranked highly in this study include economic and financial performance, particularly in the context of energy management.

## 7 Conclusions

The study found traditional challenges, such as infrastructure, finances, and human resources, still dominate organizational priorities, overshadowing explicit sustainability issues such as snow management and weather conditions. Furthermore, organizational maturity discovery may suggest that mature organizations may be better positioned to integrate sustainability practices effectively than smaller sports organizers.

This study advances theoretical understanding in the domain of sustainable sports management by refining and extending the application of institutional theory to the context of non-mega winter sports events. While institutional theory has previously been employed to explain sustainability practices among large, high-profile sports organizations (18), this study highlights the distinctive institutional environment of small and medium-sized Winter Sports Organizations (WSOs). These entities operate under lower coercive pressure, reduced visibility, and limited access to resources compared with their professional and mega event counterparts. Moreover, the observed gap between sustainability awareness and implementation is interpreted through the lens of organizational culture theory (25). This insight extends theoretical debates by foregrounding cultural inertia as a barrier to sustainability in grassroots sports, an area largely overlooked in previous studies. The study also contributes to the ongoing dialogue on environmental strategy in the context of McCullough et al.'s (108) “waves of environmentalism.” It suggests that WSOs are predominantly situated in the early or transitional phases of environmental engagement, characterized by reactive and low-intensity initiatives.

Overall, this paper contributes to theory by situating WSOs within a distinct institutional and cultural ecology, expanding sustainability studies in sports beyond elite and mega event settings, and integrating underused theoretical tools—such as organizational maturity—into the analysis of grassroots sports sustainability.

The study does have limitations. Firstly, its focus on small and medium-sized WSOs limits generalizability, especially since institutional pressures vary significantly between organizations. Moreover, the reliance on self-reported data from WSOs could introduce biases or inaccuracies in the assessment of sustainability practices and challenges. Snowball recruitment via professional referrals likely skews the sample toward more visible or better-networked organizers or the same “mind” organizations. It means it could boost (overestimate) some challenges (similar ones for the network) and underestimate others that were not significantly represented. For example selection may (1) overestimate the salience of challenges typical of more mature sustainability adopters—such as stakeholder pressure and expectations, compliance communication, and monitoring/measurement (e.g., tracking emissions an reporting to sponsors)—and (2) underestimate challenges that are more acute among less-networked organizers, such as volunteer/staff capacity limits, time and expertise gaps, and access to infrastructure or public support. Relatedly, the prevalence of formal sustainability initiatives in our data may be upwardly biased relative to the broader population of small and medium winter sports events. These considerations constrain generalizability and should be borne in mind when interpreting the relative importance of specific challenges reported. The study is also limited in its scoping review process. It is worth mentioning that the review process in both steps was limited by time till May 2024 and used limited databases (Scopus, Google Scholar). In the following study, there are some paths worth developing such as how the sustainability approach is evaluated over the years (longitudinal analysis) within changes in strategies or policies. The next limitation is the focus on open-source literature review, which may introduce bias by excluding proprietary or industry-specific sources. As a result, relevant insights from private sector reports, internal sustainability audits, or unpublished best practices—particularly those implemented by commercial sports organizations—may not be reflected. Future research could benefit from some case studies incorporating such sources to provide a more comprehensive understanding of air pollution mitigation strategies in sports event management.

Based on these limitations, future studies should include comparative analyses between small- and medium-sized WSOs and larger sports entities to provide deeper insights into institutional pressures and their effects on sustainability practices. To further develop the third research question—focused on ranking and evaluating best practices from the perspective of Winter Sports Organizations (WSOs) stakeholders—a future quantitative approach could be employed. This would involve translating insights from qualitative methods into structured survey items and testable hypotheses, enabling more robust statistical analysis. To enhance representativeness and confidence in the findings, future studies should consider probabilistic sampling techniques and the integration of multiple data sources, such as combining survey data with qualitative interviews or secondary datasets. Broader and more diverse sampling would also support deeper statistical exploration. In this study, an additional ordinary least squares (OLS) regression model was used to examine relationships between the willingness to implement sustainable practices, actual implementation, and the perceived importance of sustainability challenges.

Additionally, incorporating objective measures of sustainability performance could strengthen the reliability of findings. Future research might also explore longitudinal designs to better understand how organizational maturity influences sustainable maturity over time, providing clearer guidance for strategic sustainability integration across different types and sizes of sports organizations.

## Data Availability

The original contributions presented in the study are included in the article/Supplementary Material, further inquiries can be directed to the corresponding author.

## Appendix Examples of survey questions

“Main challenges that winter sports organizations might encounter nowadays when planning an event? Choose level, how significant is it for you (1, no significance; 5, highly significant) [Infrastructure (outdated, maintenance needed, etc.)].”

“Main challenges that winter sports organizations might encounter nowadays when planning an event? Choose level, how significant is it for you (1, no significance; 5, highly significant) [Natural resource usage].”

“Main challenges that winter sports organizations might encounter nowadays when planning an event? Choose level, how significant is it for you (1, no significance; 5, highly significant) [Sustainable transportation].”

“Main challenges that winter sports organizations might encounter nowadays when planning an event? Choose level, how significant is it for you (1, no significance; 5, highly significant) [Regulations].”

“Main challenges that winter sports organizations might encounter nowadays when planning an event? Choose level, how significant is it for you (1, no significance; 5, highly significant) [Financial resources and stability].”

“Main challenges that winter sports organizations might encounter nowadays when planning an event? Choose level, how significant is it for you (1, no significance; 5, highly significant) [Human resources].”

“Main challenges that winter sports organizations might encounter nowadays when planning an event? Choose level, how significant is it for you (1, no significance; 5, highly significant) [Weather conditions].”

“Main challenges that winter sports organizations might encounter nowadays when planning an event? Choose level, how significant is it for you (1, no significance; 5, highly significant) [Nature protection].”

“How significant is it for you to integrate more sustainable practices into winter sports? 1 (not important) 5 (very important)”

“Does your organization annually elaborate kind of an impact report of the events?”

“You've identified some challenges. Could you please briefly describe how you currently address them? (Here you can also add important challenges for you that are not mentioned among the questions before).”

## References

[1] Moon P Bayle E François A. Assessing international sport Federations’ sustainability practices: toward integrating sustainability in their main sports events. Front Sports Act Living. (2022). 10.3389/fspor.2021.752085 PMC8792860 35098117

[2] Zeimers G Anagnostopoulos C Zintz T Willem A. Organisational learning for corporate social responsibility in sport organisations. Eur Sport Manag Q. (2019) 19(1):80–101. 10.1080/16184742.2018.1546752

[3] Laing J Frost W. How green was my festival: exploring challenges and opportunities associated with staging green events. Int J Hosp Manag. (2010) 29(2):261–7. 10.1016/j.ijhm.2009.10.009

[4] Babiak K Wolfe R. Determinants of corporate social responsibility in professional sport: internal and external factors. J Sport Manag. (2009) 23(6):717–42. 10.1123/jsm.23.6.717

[5] Breitbarth T Walzel S Anagnostopoulos C van Eekeren F. Corporate social responsibility and governance in sport: “oh, the things you can find, if you don’t stay behind!”. Corp Gov. (2015) 15(2):254–73. 10.1108/CG-02-2015-0025

[6] Walters G Tacon R. The “codification” of governance in the non-profit sport sector in the UK. Eur Sport Manag Q. (2018) 18(4):482–500. 10.1080/16184742.2017.1418405

[7] Babiak K Trendafilova S. CSR and environmental responsibility: motives and pressures to adopt sustainable management practices. Corp Soc Responsib Environ Manag. (2011) 18(1):11–24. 10.1002/csr.229

[8] Trendafilova S Babiak K Heinze K. Corporate social responsibility and environmental sustainability: why professional sport is greening the playing field. Sport Manag Rev. (2013) 16(3):298–313. 10.1016/j.smr.2012.12.006

[9] Walker M Kent A Vincent J. Communicating socially responsible initiatives: an exploratory case study of a professional sport organization. Sport Mark Q. (2010) 19(4):187–95. 10.1177/106169341001900402

[10] Yoshida M Gordon BS Nakazawa M Shibuya S Fujiwara N. Bridging the gap between social media and behavioral brand loyalty. Electron Commer Res Appl. (2018) 28:208–18. 10.1016/j.elerap.2018.02.005

[11] Walker M Kent A. Do fans care? Assessing the influence of corporate social responsibility on consumer attitudes in the sport industry. J Sport Manag. (2009) 23(6):743–69. 10.1123/jsm.23.6.743

[12] Anagnostopoulos C Byers T Shilbury D. Corporate social responsibility in professional team sport organizations: towards a theory of decision-making. Eur Sport Manag Q. (2014) 14(3):259–81. 10.1080/16184742.2014.897736

[13] Weaver D Moyle BD Mclennan CL. A core/periphery perspective on mega-event sustainability: dystopic and utopic scenarios. Tour Manag. (2021) 86:104340. 10.1016/j.tourman.2021.104340

[14] Rodrigues A. The United Nations, Sport and the Environment. The New Federalist (2016). Available online at: https://www.thenewfederalist.eu/the-united-nationssport-and-the-environment

[15] International Standards Organization. (2015). ISO 20121: Event sustainability management system. Available online at: http://www.iso20121.org/ (Accessed August 20, 2024)

[16] Cognard J Berard-Chenu L Schaeffer Y François H. The snow must go on: can snowmaking keep ski resorts profitable in a changing climate? Curr Issues Tour. (2024):1–18. 10.1080/13683500.2024.2409862

[17] Steiger R Scott D. Ski tourism in a warmer world: increased adaptation and regional economic impacts in Austria. Tour Manag. (2020) 77:104032. 10.1016/j.tourman.2019.104032

[18] Zeimers G Lefebvre A Winand M Anagnostopoulos C Zintz T Willem A. Organisational factors for corporate social responsibility implementation in sport federations: a qualitative comparative analysis. Eur Sport Manag Q. (2021) 21:173–93. 10.1080/16184742.2020.1731838

[19] McCullough BP Cunningham GB. A conceptual model to understand the impetus to engage in and the expected organizational outcomes of green initiatives. Quest. (2010) 62(4):348–63. 10.1080/00336297.2010.10483654

[20] DiMaggio PJ Powell WW. The iron cage revisited: institutional isomorphism and collective rationality in organizational fields. Am Sociol Rev. (1983) 48(2):147–60. 10.2307/2095101

[21] Oliver C. The antecedents of deinstitutionalization. Organ Stud. (1992) 13:563–88. 10.1177/017084069201300403

[22] Tina Dacin M Goodstein J Richard Scott W. Institutional theory and institutional change: introduction to the special research forum. Acad Manag J. (2002) 45(1):45–56. 10.2307/3069284

[23] McCullough BP Pfahl ME Nguyen SN. The green waves of environmental sustainability in sport. Sport Soc. (2016) 19(7):1040–65. 10.1080/17430437.2015.1096251

[24] Trendafilova S Pelcher J Graham J Ziakas V. The ebbs and flows of green waves: environmental sustainability in grand slam tennis. Sport Bus Manag. (2021) 11(3):302–20. 10.1108/SBM-09-2020-0090

[25] Schein EH. Organizational Culture and Leadership. 2nd ed. San Francisco: Jossey-Bass (1992).

[26] Palter J Caraway BR. Understanding the approaches taken by private ski clubs in Southern Ontario to address climate change and sustainability. J Outdoor Recreat Tour. (2023) 43:100683. 10.1016/j.jort.2023.100683

[27] Wang W Liu Z Bu T Jiao F. Sustainable land use and green ecology: a case from the Beijing 2022 Winter Olympics venue legacy. Front Environ Sci. (2023) 11:944764. 10.3389/fenvs.2022.944764

[28] Edelman M. Sportswashing with Chinese characteristics (May 01, 2024). J Legal Aspects Sport. (2024) 34(2). 10.18060/28398

[29] Glebova E Madsen DØ. Twin transformation as a strategic approach in sport management: the synergy of digitalization and sustainability in sports. Front Sports Act Living. (2024) 8:1403793. 10.3389/fspor.2024.1403793 PMC11310929 39132514

[30] Piller S Nagel S. Environmental sustainability in Swiss sports federations—a case study on agenda setting, policy formulation, and decision-making processes. Curr Issues Sport Sci. (2023) 2:e027. 10.36950/2023.2ciss027

[31] Varriale V Cammarano A Michelino F Caputo M. The synergistic contribution of emerging technologies to sustainable development: a social network analysis. In: Taking Care of our Future: Foresight and Innovation for a Sustainable World (24th International CINet Conference; 17–19 September; Linz, Austria). Linz: CINet (2023). p. 499–515.

[32] Vrondou O Dimitropoulos P Gaitanakis L. International sports bodies application of ecological sustainability mechanisms affecting sport tourism related natural environment. Smart Tourism as a Driver for Culture and Sustainability: Fifth International Conference IACuDiT; 2018; Athens. Cham: Springer International Publishing (2019). p. 481–502

[33] McCullough BP Kellison TB. Sport ecology: conceptualizing an emerging subdiscipline within sport management. J Sport Manag. (2020) 34(6):509–19. 10.1123/jsm.2019-0186

[34] Ribeiro T Almeida V. The Rio’s transport legacy: pre-and post-games resident perceptions. Int J Sports Mark Sponsor. (2021) 22(1):32–52. 10.1108/IJSMS-04-2020-0073

[35] O’Brien D Ponting J. Sustainability and sport events: future directions and contemporary challenges. Sport Soc. (2021) 24(10):1692–708. 10.1080/17430437.2021.1913527

[36] Pereira E Trentini M Garcia L. Greenwashing in sport events: an analysis of environmental claims. Sustain J. (2022) 14(4):1171. 10.3390/su14041171

[37] Green Event Tools. (2025). Carbon footprint calculator. Available online at: https://greeneventstool.com/carbon-footprint-calculator/ (Accessed April 23, 2025).

[38] Sport Club Social Responsibility. (2025). Available online at: https://govsport.eu/projects/active-projects/page/2/ (Accessed August 20, 2025)

[39] Grix J Carmichael F. Why do governments invest in sport mega-events? A sport policy perspective. Int J Sport Policy Politics. (2020) 12(1):1–14. 10.1080/19406940.2020.1726962

[40] Preuss H. Measuring the impact of mega sport events on host economies. Event Manag. (2020) 24(6):701–14. 10.3727/152599519X15687092027039

[41] Dingle G Mallen C. Sport-environmental sustainability during COVID-19: a case study analysis. Sport Soc. (2021) 24(3):253–67. 10.1080/17430437.2021.1882715

[42] Schnitzer M Barth M. Does sport event satisfaction remain stable over time? Int J Tour Res. (2019) 21(6):785–9. 10.1002/jtr.2304

[43] Schulenkorf N Edwards D. Community development through sport events: critical perspectives. Sport Manag Rev. (2020) 23(1):9–20. 10.1016/j.smr.2019.11.004

[44] Giulianotti R. Sport mega-events, sustainability, and social impact: the ethical challenges. Ethics Sport. (2021) 48(2):119–30. 10.1177/0193723520964135

[45] Ponting J O’Brien D. Regulating “Nirvana”: sustainable surf tourism in a climate of increasing regulation. Sport Manag. Rev. (2015) 18(1):99–110. 10.1016/j.smr.2014.07.004

[46] Bernard P Chevance G Kingsbury C Baillot A Romain AJ Molinier V Climate change, physical activity, and sport: a systematic review. Sports Med. (2021) 51(5):1041–59. 10.1007/s40279-021-01439-4 33689139

[47] Casagrande Bacchiocchi S Zerbe S Cavieres LA Wellstein C. Impact of ski piste management on mountain grassland ecosystems in the Southern Alps. Sci Total Environ. (2019) 665:959–67. 10.1016/j.scitotenv.2019.02.086 30893754

[48] Gössling S Lund-Durlacher D. Tourist accommodation, climate change, and mitigation: an assessment for Austria. J Outdoor Recreat Tour. (2021) 34. 10.1016/j.jort.2021.100367

[49] Żemła M. Winter sports resorts and natural environment—systematic literature review presenting interactions between them. Sustainability. (2021). 10.3390/SU13020636

[50] Guetterman TC. Descriptions of sampling practices within five approaches to qualitative research in education and the health sciences. Qual Soc Res. (2015) 16(2):1–16. 10.17169/fqs-16.2.2290

[51] Mason M. Sample size and saturation in PhD studies using qualitative interviews. Forum. (2010) 11(3). 10.17169/fqs-11.3.1428

[52] Colasante A D'Adamo I Massis AD Italiano S. An exploratory study of stakeholder views on the sustainable development of mountain tourism. Sustain Dev. (2024). 10.1002/sd.2878

[53] Lewicki W Niekurzak M. Strategic assessment of the environmental impact of ski resorts as part of the Polish energy policy project. Energies. (2024). 10.3390/en1713316640322772

[54] Ausserladscheider V. Decoupling climate change: winter tourism and the maintenance of regional growth. New Political Econ. (2024). 10.1080/13563467.2024.2330486

[55] Uusitalo V Halonen V Koljonen H Heikkinen S Claudelin A. In search for climate neutrality in ice hockey: a case of carbon footprint reduction in a Finnish professional team. J Environ Manag. (2024). 10.1016/j.jenvman.2024.120455 38437745

[56] Zhang Y. Artificial intelligence carbon neutrality strategy in sports event management based on STIRPAT-GRU and transfer learning. Front Ecol Evol. (2023). 10.3389/fevo.2023.1275703

[57] Toscani AC Vendraminelli L Vinelli A. Environmental sustainability in the event industry: a systematic review and a research agenda. J Sustain Tour. (2024). 10.1080/09669582.2024.2309544

[58] Taheri B Thompson J. Generating socially responsible events at ski resorts. Int J Hosp Manag. (2020). 10.1016/J.IJHM.2020.102695

[59] Knowles N Scott D Rutty M. Climate change versus winter sports; can athlete climate activism change the score? Int Rev Sociol Sport. (2023). 10.1177/10126902231209226

[60] Rapley T. Sampling strategies in qualitative research. In: Flick U, editor. The Sage Handbook of Qualitative Data Analysis. London, UK: Sage (2013). Available online at: http://methods.sagepub.com/book/the-sage-handbookof-qualitative-data-analysis/n4.xml

[61] Patton MQ. Qualitative Research and Evaluation Methods. 3rd ed. Thousand Oaks CA: Sage (2002).

[62] Neuman W. Social Research Methods. Boston, MA: Pearson Education (2003).

[63] Statista (2025), All-time medal table for the Winter Olympic Games from 1924 to 2022. Available online at: https://www.statista.com/statistics/266371/winter-olympic-games-medal-tally-of-the-most-successful-nations/ (Accessed August 20, 2024)

[64] Thornberg R Charmaz K. Grounded theory and theoretical coding. In: Flick U, editor. The Sage Handbook of Qualitative Data Analysis. London, UK: Sage (2013). Available online at: http://sk.sagepub.com/reference/the-sagehandbook-of-qualitative-data-analysis/i1020.xml

[65] Moreno A Jones MJ. Impression management in corporate annual reports during the global financial crisis. Eur Manag J. (2022) 40(4):503–17. 10.1016/j.emj.2021.08.007

[66] Knowles N Scott D Steiger R. Winter sports and climate change. Sport, environment, and sustainability. Res Strat Manag. (2020):140–61.

[67] Lenzen M Sun YY Faturay F Ting YP Geschke A Malik A. The carbon footprint of global tourism. Nat Clim Change. (2018) 8(6):522–8. 10.1038/s41558-018-0141-x

[68] Steiger R Damm A Prettenthaler F Pröbstl-Haider U. Climate change and winter outdoor activities in Austria. J Outdoor Recreat Tour. (2021) 34. 10.1016/j.jort.2020.10033037519452

[69] Buerki R Elsasser H Abegg B. Climate change and winter sports: environmental and economic threats. 5th World Conference on Sport and the Environment. United Nations Environment Programme (2003). p. 1–9. Available online at: http://www.unep.org/sport_env/Documents/torinobuerki.doc

[70] Protect Our Winters EU. (2023). POW Mobility Month 2023. Available online at: https://protectourwinters.eu/pow-mobilty-month-2023/ (Accessed August 20, 2024)

[71] Kuščer K Dwyer L. Determinants of sustainability of ski resorts: do size and altitude matter? Eur Sport Manag Q. (2019) 19(4):539–59. 10.1080/16184742.2018.1550097

[72] Spandre P Morin S Lafaysse M Lejeune Y François H George-Marcelpoil E. Integration of snow management processes into a detailed snowpack model. Cold Reg Sci Technol. (2016) 125:48–64. 10.1016/j.coldregions.2016.01.002

[73] Olefs M Fischer A Lang J. Boundary conditions for artificial snow production in the Austrian Alps. J Appl Meteorol Climatol. (2010) 49(6):1096–113. 10.1175/2010JAMC2251.1

[74] Knowles N Scott D Steiger R. Sustainability of snowmaking as climate change (mal)adaptation: an assessment of water, energy, and emissions in Canada’s ski industry. Curr Issues Tour. (2024) 27(10):1613–30. 10.1080/13683500.2023.2214358

[75] Helfricht K Huss M Fischer A Otto JC. Calibrated ice thickness estimate for all glaciers in Austria. Front Earth Sci. (2019) 7:399578. 10.3389/feart.2019.00068

[76] Sommer C Malz P Seehaus TC Lippl S Zemp M Braun MH. Rapid glacier retreat and downwasting throughout the European Alps in the early 21st century. Nat Commun. (2020) 11(1). 10.1038/s41467-020-16818-0 PMC7316704 32587270

[77] Hock R Bliss A Marzeion B Giesen RH Hirabayashi Y Huss M GlacierMIP—a model intercomparison of global-scale glacier mass-balance models and projections. J Glaciol. (2019) 65(251):453–67. 10.1017/jog.2019.31

[78] FIS. (n.d.). FIS response to POW petition. Available online at: https://www.fis-ski.com/inside-fis/news/2023-24/fis-response-to-pow-petition (Accessed August 20, 2024)

[79] Salim E Ravanel L Bourdeau P Deline P. Glacier tourism and climate change: effects, adaptations, and perspectives in the Alps. Reg Environ Change. (2021) 21(4). 10.1007/s10113-021-01849-0 34776785 PMC8571665

[80] Huss M Schwyn U Bauder A Farinotti D. Quantifying the overall effect of artificial glacier melt reduction in Switzerland, 2005–2019. Cold Reg Sci Technol. (2021) 184:103237. 10.1016/j.coldregions.2021.103237

[81] Meng L Zhou Y Román MO Stokes EC Wang Z Asrar GR Artificial light at night: an underappreciated effect on phenology of deciduous woody plants. PNAS Nexus. (2022) 1(2):1–10. 10.1093/pnasnexus/pgac046 PMC9802268 36713313

[82] Lee JW. A winter sport mega-event and its aftermath: a critical review of post-Olympic PyeongChang. Local (2019) 34(7):745–52. 10.1177/0269094219889608

[83] Damm A Köberl J Prettenthaler F. Does artificial snow production pay under future climate conditions? A case study for a vulnerable ski area in Austria. Tour Manag. (2014) 43:8–21. 10.1016/j.tourman.2014.01.009

[84] Scott D Knowles N Steiger R. Is snowmaking climate change maladaptation? J Sustain Tour. (2024) 32(2):282–303. 10.1080/09669582.2022.2137729

[85] Steiger R Abegg B. The sensitivity of Austrian ski areas to climate change. Tour Plan Dev. (2013) 10(4):480–93. 10.1080/21568316.2013.804431

[86] Breiling M Charamza P. The impact of global warming on winter tourism and skiing: a regionalized model for Austrian snow conditions. Reg Environ Change. (1999) 1(1):4–14. 10.1007/s101130050003

[87] Steiger R. The impact of climate change on ski season length and snowmaking requirements in Tyrol, Austria. Clim Res. (2010) 43(3):251–62. 10.3354/cr00941

[88] Skiresort.de. (n.d.). Das Weltgrößte Testportal von Skigebiete.n. Available online at: https://www.skiresort.de/ (Accessed August 20, 2024)

[89] Witting M Schmude J. Impacts of climate and demographic change on future skier demand and its economic consequences: evidence from a ski resort in the German Alps. J Outdoor Recreat Tour. (2019) 26:50–60. 10.1016/j.jort.2019.03.002

[90] Roth R Schiefer D Siller HJ Beyer J Fehringer A Bosio B (2016). The future of winter travelling in the Alps: Zukunft Wintersport Alpen. Available online at: https://fis.dshs-koeln.de/en/publications/the-future-of-winter-travelling-in-the-alps-zukunft-wintersport-a (Accessed August 13, 2024)

[91] Chawla L. Childhood nature connection and constructive hope: a review of research on connecting with nature and coping with environmental loss. People Nat. (2020) 2(3):619–42. 10.1002/pan3.10128

[92] Goldman D Alkaher I. Outdoor environmental education: grounding a tradition within environmental education. In: International Explorations in Outdoor and Environmental Education. Melbourne: Springer (2023). p. 11–32. 10.1007/978-3-031-29257-6_2

[93] Federació Catalana d’Esports d’Hivern. (n.d.). Esport Blanc Escolar (EBE). Available online at: https://www.fceh.cat/esport-blanc-escolar-ebe/ (Accessed August 13, 2024)

[94] Frühauf A Niedermeier M Kopp M. Intention to engage in winter sport in climate change affected environments. Front Public Health. (2020) 8. 10.3389/fpubh.2020.594993 PMC7775576 33392137

[95] Scott D Steiger R Rutty M Fang Y. The changing geography of the Winter Olympic and Paralympic Games in a warmer world. Curr Issues Tour. (2019) 22(11):1301–11. 10.1080/13683500.2018.1436161

[96] Horne J Manzenreiter W. An introduction to the sociology of sports mega-events. Sociol Rev. (2006) 54(Suppl. 2):1–24. 10.1111/j.1467-954X.2006.00650.x

[97] Gollagher P Fastenrath S. Transformative climate resilience and sport mega-events: the case of the Australian Open. Environ Innov Soc Trans. (2023) 48:100762. 10.1016/j.eist.2023.100762

[98] Van den Hurk M Verhoest K. The governance of public–private partnerships in sports infrastructure: interfering complexities in Belgium. Int J Proj Manag. (2015) 33(1):201–11. 10.1016/j.ijproman.2014.05.005

[99] Cayolla RR Quintela JA Santos T. “If you don’t know me by now”—the importance of sustainability initiative awareness for stakeholders of professional sports organizations. Sustainability. (2022) 14(9):4917. 10.3390/su14094917

[100] Kellison TB Kim YK. Marketing pro-environmental venues in professional sport: planting seeds of change among existing and prospective consumers. J Sport Manag. (2014) 28(1):34–48. 10.1123/jsm.2011-0127

[101] Hugaerts I Scheerder J Zeimers G Corthouts J Van de Sype C Könecke T. Are sport organisations environmentally sustainable?—a website analysis of sport federations in Belgium. Eur Sport Manag Q. (2022) 23(1):38–58. 10.1080/16184742.2022.2093391

[102] Buckley RC Pickering CM Warnken J. Environmental management for alpine tourism and resorts in Australia. In: Godde P Price M Zimmerman F, editors. Tourism and Development in Mountain Regions. New York, NY: CABI (2000). p. 27–45.

[103] Wheeler K Nauright J. A global perspective on the environmental impact of golf. Sport Soc. (2006) 9(3):427–43. 10.1080/17430430600673449

[104] Porter M Reinhardt F. A strategic approach to climate change. Harv Bus Rev. (2007) 85(10):22–6.

[105] Aragón-Correa JA Sharma S. A contingent resource-based view of proactive corporate environmental strategy. Acad Manag Rev. (2003) 28(1):71–88. 10.5465/amr.2003.8925233

[106] McCullough BP Cunningham GB. Recycling intentions among youth baseball spectators. Int J Sport Manag Mark. (2011) 10(1–2):104–20. 10.1504/IJSMM.2011.043618

[107] Pfahl M. Sport & the Natural Environment: A Strategic Guide. Kendall Hunt Publishing Company (2011).

[108] McCullough BP Pfahl ME Nguyen SN. The green waves of environmental sustainability in sport. Sport Soc. (2015) 19(7):1040–65. 10.1080/17430437.2015.1096251

[109] Albert M Bauche X Closmann L Eichholz T Preis K. Sustainability management in non-governmental organisations: development of a maturity model. Int J Innov Sustain Dev. (2022) 16(3–4):425–60. 10.1504/IJISD.2022.123905

[110] Odważny F Wojtkowiak D Cyplik P Adamczak M. Concept for measuring organizational maturity supporting sustainable development goals. LogForum. (2019) 15(2):237–47. 10.17270/J.LOG.2019.321

[111] Daddi T Rizzi F Pretner G Todaro N Annunziata E Frey M Environmental management of sport events: a focus on European professional football. Sport Bus Manag. (2022) 12(2):208–32. 10.1108/SBM-05-2020-0046

[112] World. (2020). Sustainability at World Athletics. Available online at: https://worldathletics.org/athletics-better-world/sustainability (Accessed August 20, 2025)

[113] Chen C Kellison T. The clock is ticking: contexts, tensions and opportunities for addressing environmental justice in sport management. Sport Bus Manag. (2023) 13(3):376–96. 10.1108/SBM-08-2022-0071

[114] Eusébio C Carneiro MJ Madaleno M Robaina M Rodrigues V Russo M The impact of air quality on tourism: a systematic literature review. J Tour Fut. (2021) 7(1):111–30. 10.1108/JTF-06-2019-0049

[115] Bouquet P Molinari A Bortolan L Dal Prà A Bezzi G Vettorato S. A cloud-based management system for a data infrastructure in four winter sport facilities. Star. (2022) 4(3):295–306. 10.1109/STAR53492.2022.9859651

[116] International Report on Snow & Mountain Tourism. (2024). Available online at: https://vanat.ch/international-report-on-snow-mountain-tourism.shtml (Accessed August 20, 2024)

