# Correction: Are we able to have sustainable winter sports events? Main challenges and future directions

**DOI:** 10.3389/fspor.2026.1855984

**Published:** 2026-05-19

**Authors:** Igor Perechuda

**Affiliations:** 1 Department of Management, LUNEX University of Applied Sciences, Differdange, Luxembourg; 2 Luxembourg Health & Sport Sciences Research Institute A.s.b.l., Differdange, Luxembourg

**Keywords:** governance, sustainability, management, event, winter, sports

A Correction on Are we able to have sustainable winter sports events? Main challenges and future directions Perechuda I (2025). Front Sports Act. Living 7:1605544. doi: 10.3389/fspor.2025.1605544

An incorrect **Funding** statement was provided:

“The author declares that financial support was received for the research and/or publication of this article. Study based on EU project number 101133676, ERASMUS-SPORT-2023-SCP.”

The correct **Funding** statement reads:

“The author declares that financial support was received for the research and/or publication of this article. The study is based on EU project number 101133676, ERASMUS-SPORT-2023-SCP, entitled European Week of Winter Sport. The project is supported by the consortium partners: L'ORMA (Paolo Menescardi, Sara Brivio), Kilian Jornet Foundation (Laura Viñals Rotellar, Anna González Manjón), EUSA (Andrej Pisl), Ski klub Rijeka (Natalija Šikić, Sebastian Brigović), NGO NEST Berlin (Christian Porstner, Roberto Solinas), Deporte para la Educación y la Salud (Andreu Raya-Demidoff, Pablo Raya-Demidoff, Aitor Ortuzar, Gustavo Peña, Eduardo Dobon, Tommy García), European Platform for Sport Innovation (Matias Pagura), LUNEX (Igor Perechuda, Mara Wagner) and the Bulgarian Sports Development Association (Yoanna Dochevska, Ivaylo Zdravkov).”

The original version of this article has been updated.

